# Ethnic comparison in takotsubo syndrome: novel insights from the International Takotsubo Registry

**DOI:** 10.1007/s00392-021-01857-4

**Published:** 2021-05-19

**Authors:** Yoichi Imori, Ken Kato, Victoria L. Cammann, Konrad A. Szawan, Manfred Wischnewsky, Sara Dreiding, Michael Würdinger, Maximilian Schönberger, Vanya Petkova, David Niederseer, Rena A. Levinson, Davide Di Vece, Sebastiano Gili, Burkhardt Seifert, Masaki Wakita, Noriko Suzuki, Rodolfo Citro, Eduardo Bossone, Susanne Heiner, Maike Knorr, Thomas Jansen, Thomas Münzel, Fabrizio D’Ascenzo, Jennifer Franke, Ioana Sorici-Barb, Hugo A. Katus, Annahita Sarcon, Jerold Shinbane, L. Christian Napp, Johann Bauersachs, Milosz Jaguszewski, Reiko Shiomura, Shunichi Nakamura, Hitoshi Takano, Michel Noutsias, Christof Burgdorf, Iwao Ishibashi, Toshiharu Himi, Wolfgang Koenig, Heribert Schunkert, Holger Thiele, Behrouz Kherad, Carsten Tschöpe, Burkert M. Pieske, Lawrence Rajan, Guido Michels, Roman Pfister, Shingo Mizuno, Alessandro Cuneo, Claudius Jacobshagen, Gerd Hasenfuß, Mahir Karakas, Hiroki Mochizuki, Alexander Pott, Wolfgang Rottbauer, Samir M. Said, Ruediger C. Braun-Dullaeus, Adrian Banning, Toshiaki Isogai, Akihisa Kimura, Florim Cuculi, Richard Kobza, Thomas A. Fischer, Tuija Vasankari, K. E. Juhani Airaksinen, Yasuhiro Tomita, Monika Budnik, Grzegorz Opolski, Rafal Dworakowski, Philip MacCarthy, Christoph Kaiser, Stefan Osswald, Leonarda Galiuto, Filippo Crea, Wolfgang Dichtl, Tsutomu Murakami, Yuji Ikari, Klaus Empen, Daniel Beug, Stephan B. Felix, Clément Delmas, Olivier Lairez, Tetsuo Yamaguchi, Ibrahim El-Battrawy, Ibrahim Akin, Martin Borggrefe, John D. Horowitz, Martin Kozel, Petr Tousek, Petr Widimský, Ekaterina Gilyarova, Alexandra Shilova, Mikhail Gilyarov, Michael Neuhaus, Philippe Meyer, Jose David Arroja, Christina Chan, Paul Bridgman, Jan Galuszka, Gregor Poglajen, Pedro Carrilho-Ferreira, Fausto J. Pinto, Christian Hauck, Lars S. Maier, Kan Liu, Carlo Di Mario, Carla Paolini, Claudio Bilato, Matteo Bianco, Lucas Jörg, Hans Rickli, David E. Winchester, Christian Ukena, Michael Böhm, Jeroen J. Bax, Abhiram Prasad, Charanjit S. Rihal, Shigeru Saito, Yoshio Kobayashi, Thomas F. Lüscher, Frank Ruschitzka, Wataru Shimizu, Jelena R. Ghadri, Christian Templin

**Affiliations:** 1 Department of Cardiovascular Medicine, Nippon Medical School, Tokyo, Japan; 2 Department of Cardiology, University Heart Center, University Hospital Zurich, Raemistrasse 100, 8091 Zurich, Switzerland; 3 Department of Cardiovascular Medicine, Chiba University Graduate School of Medicine, Chiba, Japan; 4 Department of Mathematics and Computer Science, University of Bremen, Bremen, Germany; 5 Centro Cardiologico Monzino, IRCCS, Milan, Italy; 6 Department of Biostatistics, Epidemiology, Biostatistics and Prevention Institute, University of Zurich, Zurich, Switzerland; 7 Heart Department, University Hospital “San Giovanni di Dio E Ruggi d’Aragona”, Salerno, Italy; 8 Division of Cardiology, “Antonio Cardarelli” Hospital, Naples, Italy; 9 Center for Cardiology, University Medical Center Mainz, Cardiology 1, Mainz, Germany; 10 Division of Cardiology, Department of Medical Sciences, AOU Citta Della Salute e Della Scienza, University of Turin, Turin, Italy; 11 Department of Cardiology, Medical University Hospital Heidelberg, Heidelberg, Germany; 12 Section of Cardiac Electrophysiology, Department of Medicine, University of California-San Francisco, San Francisco, CA USA; 13 Keck School of Medicine, University of Southern California, Los Angeles, CA USA; 14 Department of Cardiology and Angiology, Hannover Medical School, Hannover, Germany; 15 First Department of Cardiology, Medical University of Gdansk, Gdansk, Poland; 16 Department of Internal Medicine III, Division of Cardiology, Angiology and Intensive Medical Care, University Hospital Halle, Martin-Luther-University Halle-Wittenberg, Halle (Saale), Germany; 17 Heart and Vascular Centre Bad Bevensen, Bad Bevensen, Germany; 18 Department of Cardiology, Chiba Emergency Medical Center, Chiba, Japan; 19 Division of Cardiology, Kimitsu Central Hospital, Kisarazu, Japan; 20 Deutsches Herzzentrum München, Technische Universität München, Munich, Germany; 21 DZHK (German Centre for Cardiovascular Research), Partner Site Munich Heart Alliance, Munich, Germany; 22 Department of Internal Medicine/Cardiology, Heart Center Leipzig - University Hospital, Leipzig, Germany; 23 Department of Cardiology, Charité-Universitätsmedizin Berlin, Berlin, Germany; 24 TJ Health Partners Heart and Vascular, Glasgow, KY USA; 25 Department of Internal Medicine III, Heart Center University of Cologne, Cologne, Germany; 26 Department of Cardiology & Catheterization Laboratories, Shonankamakura General Hospital, Kamakura, Japan; 27 Krankenhaus “Maria Hilf“ Medizinische Klinik, Stadtlohn, Germany; 28 Clinic for Cardiology and Pneumology, Goettingen University, Goettingen, Germany; 29 Department of General and Interventional Cardiology, University Heart Center Hamburg, Hamburg, Germany; 30 DZHK (German Centre for Cardiovascular Research), partner site Hamburg/Kiel/Luebeck, Hamburg, Germany; 31 Department of Cardiology, St. Luke’s International Hospital, Tokyo, Japan; 32 Department of Internal Medicine II – Cardiology, Medical Center, University of Ulm, Ulm, Germany; 33 Department of Cardiac Electrophysiology, Helios St. Marienberg Hospital Helmstedt, Helmstedt, Germany; 34 Internal Medicine/Cardiology, Angiology, and Pneumology, Magdeburg University, Magdeburg, Germany; 35 Department of Cardiology, John Radcliffe Hospital, Oxford University Hospitals, Oxford, UK; 36 Department of Cardiology, Tokyo Metropolitan Tama Medical Center, Tokyo, Japan; 37 Department of Cardiology, Kantonsspital Lucerne, Lucerne, Switzerland; 38 Department of Cardiology, Kantonsspital Winterthur, Winterthur, Switzerland; 39 Heart Center, Turku University Hospital and University of Turku, Turku, Finland; 40 Department of Cardiology, Toranomon Hospital, Tokyo, Japan; 41 Department of Cardiology, Medical University of Warsaw, Warsaw, Poland; 42 Department of Cardiology, King’s College Hospital, London, UK; 43 Department of Cardiology, University Hospital Basel, Basel, Switzerland; 44 Fondazione Policlinico Universitario A. Gemelli IRCCS, Roma, Italia; 45 University Hospital for Internal Medicine III (Cardiology and Angiology), Medical University Innsbruck, Innsbruck, Austria; 46 Department of Cardiology, Tokai University School of Medicine, Isehara, Japan; 47 Department of Internal Medicine, Kreiskrankenhaus Wolgast, Wolgast, Germany; 48 Department of Internal Medicine B, University Medicine Greifswald, Greifswald, Germany; 49 DZHK (German Centre for Cardiovascular Research), partner site Greifswald, Greifswald, Germany; 50 Department of Cardiology and Cardiac Imaging Center, University Hospital of Rangueil, Toulouse, France; 51 Department of Cardiology, Japanese Red Cross Musashino Hospital, Tokyo, Japan; 52 First Department of Medicine, Faculty of Medicine, University Medical Centre Mannheim (UMM) University of Heidelberg, Mannheim, Germany; 53 DZHK (German Center for Cardiovascular Research), Partner Site, Heidelberg-Mannheim, Mannheim, Germany; 54 Department of Cardiology, Basil Hetzel Institute, Queen Elizabeth Hospital, University of Adelaide, Adelaide, Australia; 55 Cardiocenter, Third Faculty of Medicine, Charles University Prague, Prague, Czech Republic; 56 Intensive Coronary Care Unit, Moscow City Hospital # 1 Named After N. Pirogov, Moscow, Russia; 57 Department of Cardiology, Kantonsspital Frauenfeld, Frauenfeld, Switzerland; 58 Service de Cardiologie, Hôpitaux Universitaires de Genève, Geneva, Switzerland; 59 Department of Cardiology, Christchurch Hospital, Christchurch, New Zealand; 60 Department of Internal Medicine I - Cardiology, University Hospital Olomouc, Olomouc, Czech Republic; 61 Advanced Heart Failure and Transplantation Center, University Medical Center Ljubljana, Ljubljana, Slovenia; 62 Santa Maria University Hospital, CHULN Center of Cardiology of the University of Lisbon, Lisbon School of Medicine, Lisbon Academic Medical Center, Lisboa, Portugal; 63 Universitäres Herzzentrum Regensburg, Regensburg, Germany; 64 Division of Cardiology, Heart and Vascular Center, University of Iowa, Iowa City, IA USA; 65 Structural Interventional Cardiology, Careggi University Hospital, Florence, Italy; 66 Local Health Unit n.8, Cardiology Unit, Arzignano, Vicenza, Italy; 67 Division of Cardiology, A.O.U San Luigi Gonzaga, Orbassano, Turin, Italy; 68 Department of Cardiology, Kantonsspital St. Gallen, St. Gallen, Switzerland; 69 Division of Cardiovascular Medicine, Department of Medicine, College of Medicine, University of Florida, Gainesville, FL USA; 70 Klinik Für Innere Medizin III, Universitätsklinikum des Saarlandes, Homburg Saar, Germany; 71 Department of Cardiology, Leiden University Medical Centre, Leiden, The Netherlands; 72 Department of Cardiovascular Diseases, Mayo Clinic, Rochester, MN USA; 73 Center for Molecular Cardiology, Schlieren Campus, University of Zurich, Zurich, Switzerland; 74 Royal Brompton and Harefield Hospitals Trust and Imperial College, London, UK

**Keywords:** Takotsubo syndrome, Broken heart syndrome, Ethnicity, Race

## Abstract

**Background:**

Ethnic disparities have been reported in cardiovascular disease. However, ethnic disparities in takotsubo syndrome (TTS) remain elusive. This study assessed differences in clinical characteristics between Japanese and European TTS patients and determined the impact of ethnicity on in-hospital outcomes.

**Methods:**

TTS patients in Japan were enrolled from 10 hospitals and TTS patients in Europe were enrolled from 32 hospitals participating in the International Takotsubo Registry. Clinical characteristics and in-hospital outcomes were compared between Japanese and European patients.

**Results:**

A total of 503 Japanese and 1670 European patients were included. Japanese patients were older (72.6 ± 11.4 years vs. 68.0 ± 12.0 years; *p* < 0.001) and more likely to be male (18.5 vs. 8.4%; *p* < 0.001) than European TTS patients. Physical triggering factors were more common (45.5 vs. 32.0%; *p* < 0.001), and emotional triggers less common (17.5 vs. 31.5%; *p* < 0.001), in Japanese patients than in European patients. Japanese patients were more likely to experience cardiogenic shock during the acute phase (15.5 vs. 9.0%; *p* < 0.001) and had a higher in-hospital mortality (8.2 vs. 3.2%; *p* < 0.001). However, ethnicity itself did not appear to have an impact on in-hospital mortality. Machine learning approach revealed that the presence of physical stressors was the most important prognostic factor in both Japanese and European TTS patients.

**Conclusion:**

Differences in clinical characteristics and in-hospital outcomes between Japanese and European TTS patients exist. Ethnicity does not impact the outcome in TTS patients. The worse in-hospital outcome in Japanese patients, is mainly driven by the higher prevalence of physical triggers.

**Trial Registration:**

URL: https://www.clinicaltrials.gov; Unique Identifier: NCT01947621.

**Supplementary Information:**

The online version contains supplementary material available at 10.1007/s00392-021-01857-4.

## Introduction

Takotsubo syndrome (TTS) is characterized by acute left ventricular (LV) systolic dysfunction, which is often triggered by emotional or physical stressors [1]. The condition was first reported from Japan in 1990 and the terminology derived from a Japanese octopus trap known as takotsubo because the LV in TTS mimics the shape of it [2, 3]. TTS was largely unknown outside Japan until the first reports from Europe and the US were published in the late 90 s [4, 5]. Since then, TTS has been reported from all continents, in both sexes and in different ethnic groups [6–11]. Although TTS was initially considered to be harmless, it is now known that it has a wide range of presentations and outcomes, depending on the triggering factor [12]. It may even be associated with life-threatening complications, with outcomes comparable to those of acute coronary syndromes [12–14].

Ethnic and geographic disparities are known to exist in many cardiovascular diseases [15, 16]. These differences may be related to socioeconomic factors, healthcare systems, or genetic background, but there is no clear evidence in support of such speculation. To date, no large head-to-head comparison between different ethnic and geographic groups has been conducted in the field of TTS research. Little is known about ethnic differences in outcomes in TTS, especially between Asian and European patients [17].

The present study aimed to investigate differences in clinical characteristics and in-hospital outcomes between Japanese and European TTS patients and to evaluate the impact of ethnic background on outcomes.

## Methods

### Patients and inclusion criteria

**Fig. 1:**
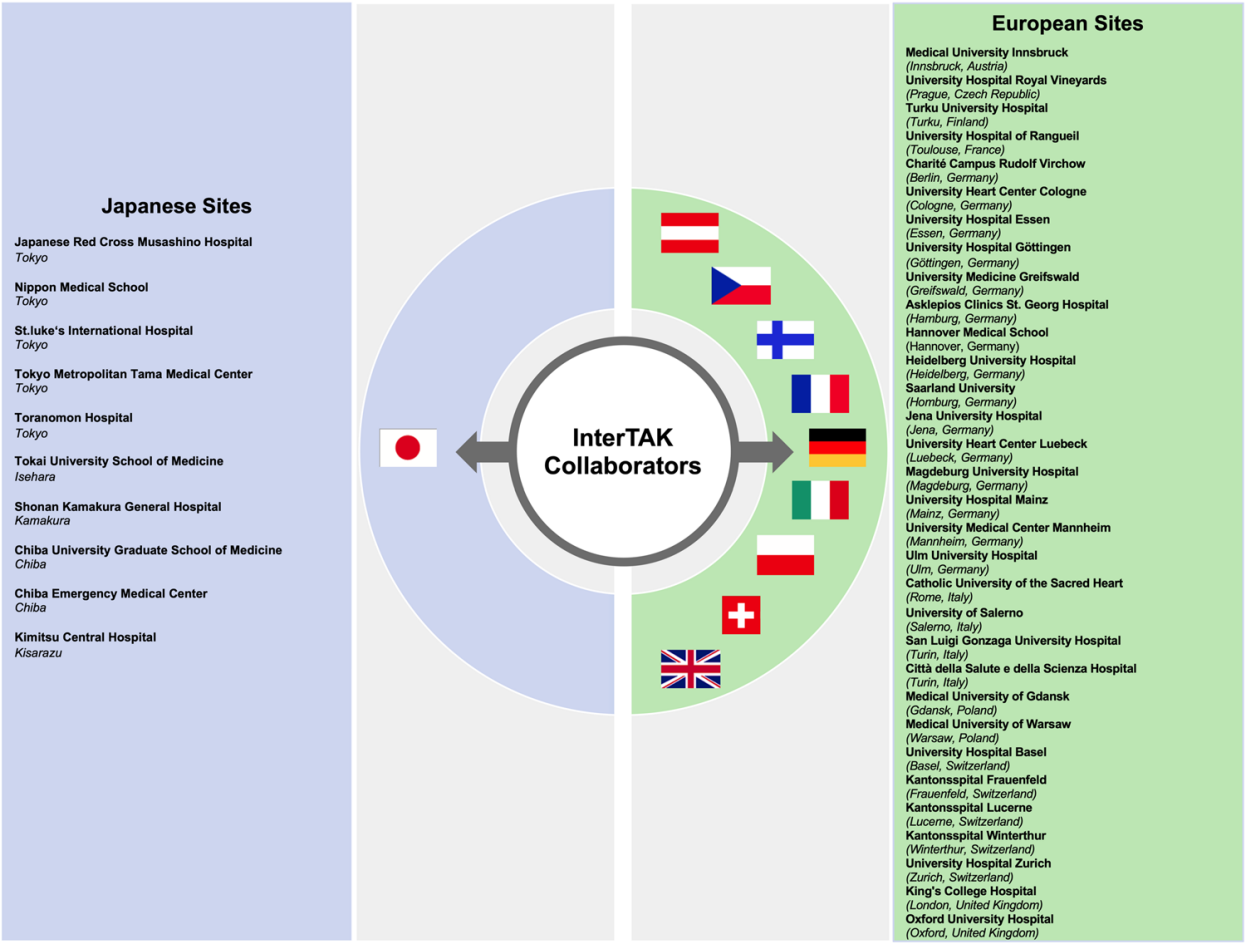
Collaborators from Japan and Europe. Participants in this study included 10 centers from Japan and 32 centers from 9 countries in Europe (including Austria, Czech Republic, Finland, France, Germany, Italy, Poland, Switzerland, and the UK)

The Japanese TTS patients were enrolled from 10 different hospitals, while the European TTS patients were enrolled from the University Hospital Zurich and 31 other collaborating hospitals in 9 countries (Austria, Czech Republic, Finland, France, Germany, Italy, Poland, Switzerland, and the UK) (Fig. 1). Data were collected from January 1, 2011, through October 31, 2017, and patients with unknown ethnicity were excluded from the present analysis. All patients were diagnosed according to the InterTAK Diagnostic Criteria [18]:.

1. Transient LV wall motion abnormality presenting as apical ballooning or midventricular, basal, or focal wall motion abnormalities. The wall motion abnormality usually extends beyond a single epicardial coronary artery distribution; however, in rare cases, especially in focal TTS, wall motion abnormality may be limited to a single coronary artery distribution. TTS patients who died during the acute phase, before complete recovery of LV wall motion, were also eligible for inclusion.
2. TTS event is typically triggered by emotional or physical, or combined stress. However, this is not obligatory. Neurologic disorders or pheochromocytoma may also serve as triggers.
3. Appearance of new electrocardiographic (ECG) abnormalities. Occasionally, there may not be any electrocardiographic changes.
4. Occurrence of moderate elevations of cardiac biomarkers (troponin and/or creatine kinase). Elevation of brain natriuretic peptide is common.
5. Coexisting significant coronary artery disease, which is not related to the wall motion abnormality, does not rule out TTS.
6. No evidence of infectious myocarditis.

The study protocol was approved by the respective local ethics committees or institutional review boards at each collaboration site. Due to the, in part, retrospective nature of the study, the ethics committees of most study centers waived the need for informed consent. At centers where the ethics committees or institutional review boards required informed consent, formal written consent was obtained from all included patients or their surrogates.

### Clinical characteristics and in-hospital outcomes

We compared demographic data, clinical characteristics, triggering factors, taktosubo type, laboratory values, ECG findings, vital signs, hemodynamic findings, cardiovascular risk factors, and medications prescribed (before admission and at discharge) between the Japanese and European TTS cohorts. Acute cardiac care treatment (use of catecholamine and intra-aortic balloon pump [IABP]) and in-hospital complications (cardiogenic shock and death) were also compared between the two groups.

### Statistical analysis

Continuous data were summarized as means ± standard deviations or medians and interquartile ranges. Categorical variables were summarized as numbers and percentages. Comparisons of characteristics between different groups were performed using the unpaired *t* test or the Mann–Whitney *U* test for continuous variables and the Pearson chi-squared test for categorical variables. Variables that were statistically significant on univariate analysis were included in multivariate models, using in-hospital mortality as an endpoint. Two different models (Model I: without ethnicity as a covariate and Model II with ethnicity as a covariate) were used. The Hosmer–Lemeshow test for goodness of fit was performed for both logistic regression models. To compare these two models, and to clear any doubts about which model is more appropriate, three different statistical approaches were used. First, the Brier score was used to quantify the accuracy of risk predictions of our two logistic regression models by comparing predicted risks with observed outcomes at the individual level. The lower the Brier score is for a set of predictions, the better the predictions are calibrated. Second, classification performance was evaluated by use of receiver operating characteristic (ROC) curves. Third, the predictiveness curve was calculated [19]. The predictiveness curve is a plot of the cumulative percentage of individuals to the predicted risks. The cumulative percentage indicates the percentage of individuals with predicted risk equal to, or lower than, the risk value. The predictiveness curve also displays essential information about risk that is not displayed by the ROC curve [19]. The Delong [20] as well as the Venkatraman test [21] were used to compare the AUCs for equivalence (equivalence bound 5%) and then for equality ((AUCII—AUCI) = 0) (condition = in-hospital death). To evaluate the influence of each factor on in-hospital death, we used a radial basis function network (RBF-net) [22]. RBF networks are artificial neural networks that use radial basis functions as activation functions. They are primarily used for regression or function approximation. The Bayesian Information Criterion (BIC) determines the number of units in the hidden layer. The "best" number of hidden units is the one that yields the smallest BIC in the training data. We used normalized Gaussian radial basis functions as activation functions for the hidden layer. For the output layer, we used the identity function as the activation function; thus, the output units are simply weighted sums of the hidden units. The output of the network (in-hospital death) is, therefore, a linear combination of normalized Gaussian radial basis functions of the inputs and neuron parameters. Decision trees such as Classification And Regression Trees (CART) or exhaustive CHi-squared Automatic Interaction Detector (CHAID), were used in addition as a nonparametric supervised learning method for classification and regression. Decision trees are adaptive in at least three aspects: they (1) adapt to favorable conditions near the Bayes decision boundary; (2) focus on data distributed on lower dimensional manifolds, and (3) reject irrelevant features.

All tests were two sided and statistical significance was defined as *p* < 0.05. Statistical analyses were performed using IBM SPSS Statistics, version 25.0 (IBM Corp., Armonk, NY, USA) and R, version 3.5 (https://cran.r-project.org/).

## Results

A total of 503 Japanese TTS patients and 1670 European TTS patients were included in this study. Table 1 presents a comparison of clinical characteristics between Japanese and European patients. The proportion of male patients was significantly higher in the Japanese cohort (18.5 vs. 8.4%; *p* < 0.001). Japanese patients were older (72.6 ± 11.4 vs. 68.0 ± 12.0 years; *p* < 0.001) and had lower body mass index (21.0 ± 3.6 vs. 24.8 ± 4.6 kg/m^2^; p < 0.001) than European patients. Japanese patients had lower prevalence of hypertension (52.7 vs. 66.3%; *p* < 0.001), current smoking (15.2 vs. 18.7%; *p* < 0.001), and higher prevalence of diabetes mellitus (18.1 vs. 13.5%, *p* = 0.011) than European patients. Troponin (median factor increase of the upper limit of normal, 16.78 vs. 7.67; *p* < 0.001) and brain natriuretic peptide plasma levels (median factor increase of the upper limit of normal, 13.14 vs. 5.72; *p* < 0.001) at hospital admission were significantly higher in Japanese patients. ST-segment elevation on admission was more common in Japanese patients than in European patients (70.3 vs. 45.0%; *p* < 0.001). Left ventricular ejection fraction (LVEF) on admission was significantly higher in Japanese than in European patients (44.5 ± 13.3 vs. 41.8% ± 11.2%; *p* < 0.001). Japanese patients were more likely to have physical triggers (45.5 vs. 32.0%; *p* < 0.001), and less likely to have emotional triggers (17.5 vs. 31.5%; *p* < 0.001) than European patients. Details on triggering factors are shown in Supplementary Fig. 1. Before admission, Japanese patients were more likely to be using calcium-channel antagonists (25.5 vs. 7.2%; *p* < 0.001), while European patients were more likely to be using angiotensin-converting enzyme inhibitors/angiotensin-receptor blockers (39.4 vs. 30.8%; *p* = 0.001) and beta-blockers (30.9 vs. 7.9%; *p* < 0.001). The same trend in medications prescribed was seen at discharge (Table 1).

**Table 1:** Comparisons of clinical characteristics between Japanese and European patients with TTS

Characteristic	Japanese patients	European patients	*p* value
	*n* = 503	*n* = 1670	
Demographics			
Female sex	410 (81.5)	1529 (91.6)	< 0.001
Age, mean (SD), y	72.6 (11.4), *n* = 503	68.0 (12.0), *n* = 1670	< 0.001
BMI, mean (SD), kg/m^2^	21.0 (3.6), *n* = 470	24.8 (4.6), *n* = 1239	< 0.001
Triggers			
Physical trigger	229 (45.5)	534 (32.0)	< 0.001
Emotional trigger	88 (17.5)	526 (31.5)	< 0.001
Both emotional and physical trigger	8 (1.6)	122 (7.3)	< 0.001
No evident trigger	178 (35.4)	488 (29.2)	0.009
Takotsubo type			
Apical type	358 (71.2)	1169 (70.0)	0.61
Cardiac biomarkers on admission, median (IQR)			
Troponin, fold ULN*	16.78 (4.58–48.94), *n* = 337	7.67 (2.50–21.15), *n* = 1233	< 0.001
Creatine kinase, fold ULN	0.96 (0.47–1.83), *n* = 484	0.87 (0.56–1.45), *n* = 1204	0.62
BNP, fold ULN†	13.14 (4.79–35.41), *n* = 321	5.72 (2.11–15.34), *n* = 414	< 0.001
Inflammatory markers on admission, median (IQR)			
CRP, mg/L	5.15 (1.09–29.40), *n* = 476	4.00 (1.50–12.30), *n* = 1215	0.026
WBC, 10^3^ /µL	9.00 (6.70–11.85), *n* = 497	9.60 (7.44–12.27), *n* = 1390	0.001
ECG on admission			
ST-segment elevation	353 of 502 (70.3)	676 of 1503 (45.0)	< 0.001
Vital signs, mean (SD**)**			
Heart rate, beats/min	92.8 (21.8), *n* = 398	85.9 (20.0), *n* = 1319	< 0.001
Systolic blood pressure, mm Hg	130.9 (30.7), *n* = 409	132.4 (28.1), *n* = 1339	0.36
Diastolic blood pressure, mm Hg	77.6 (20.0), *n* = 404	77.2 (15.7), *n* = 1325	0.68
Hemodynamics, mean ± SD			
Left ventricular ejection fraction, %^‡^	44.5 (13.3), *n* = 471	41.8 (11.2), *n* = 1485	< 0.001
Cardiovascular risk factors			
Hypertension	265 of 503 (52.7)	1078 of 1627 (66.3)	< 0.001
Current smoking	75 of 494 (15.2)	289 of 1548 (18.7)	< 0.001
Diabetes mellitus	91 of 503 (18.1)	222 of 1642 (13.5)	0.011
Hypercholesterolemia	169 of 503 (33.6)	506 of 1594 (31.7)	0.44
Medication on admission			
Cardiovascular medication			
ACE-inhibitor or ARB	147 of 478 (30.8)	500 of 1268 (39.4)	0.001
Beta-blocker	38 of 478 (7.9)	392 of 1268 (30.9)	< 0.001
Calcium-channel antagonist	122 of 478 (25.5)	89 of 1236 (7.2)	< 0.001
Statin	80 of 478 (16.7)	214 of 1236 (17.3)	0.78
Medication at discharge			
Cardiovascular medication			
ACE-inhibitor or ARB	199 of 448 (44.4)	1190 of 1471 (80.9)	< 0.001
Beta-blocker	120 of 448 (26.8)	1153 of 1471 (78.4)	< 0.001
Calcium-channel antagonist	125 of 462 (27.1)	134 of 1471 (9.1)	< 0.001
Statin	112 of 448 (25.0)	749 of 1471 (50.9)	< 0.001
Acute cardiac care treatment			
Catecholamine use	71 of 502 (14.1)	170 of 1653 (10.3)	0.016
Intra-aortic balloon pump	28 of 500 (5.6)	35 of 1652 (2.1)	< 0.001
In-hospital complications			
Cardiogenic shock	78 of 502 (15.5)	149 of 1660 (9.0)	< 0.001
Death	41 (8.2)	53 (3.2)	< 0.001

*ACE* angiotensin-converting-enzyme, *ARB* angiotensin-receptor blocker, *BMI*  body mass index, *BNP* brain natriuretic peptide, *CRP* C-reactive protein, *ECG* electrocardiogram, *IQR* interquartile range, *SD* standard deviation, *TTS* takotsubo syndrome, *ULN* upper limit of the normal, *WBC* white blood cell count

Data are presented as number (percentage) of patients unless otherwise indicated

*Including upper limits of the normal range for troponin T, high-sensitivity troponin T, and troponin I

^†^Including upper limits of the normal range for brain natriuretic peptide and the N-terminal of prohormone brain natriuretic peptide

^‡^Data obtained during catheterization or echocardiography; if both results were available data from catheterization were used

While in-hospital Japanese patients were more likely to experience cardiogenic shock (15.5 vs. 9.0%; *p* < 0.001) and more likely to receive treatment with catecholamines (14.1 vs. 10.3%; *p* = 0.016) or IABP (5.6 vs. 2.1%; *p* < 0.001) to maintain hemodynamic stability. In-hospital mortality was significantly higher in Japanese patients than in European patients (8.2 vs. 3.2%; *p* < 0.001).

**Fig. 2:**
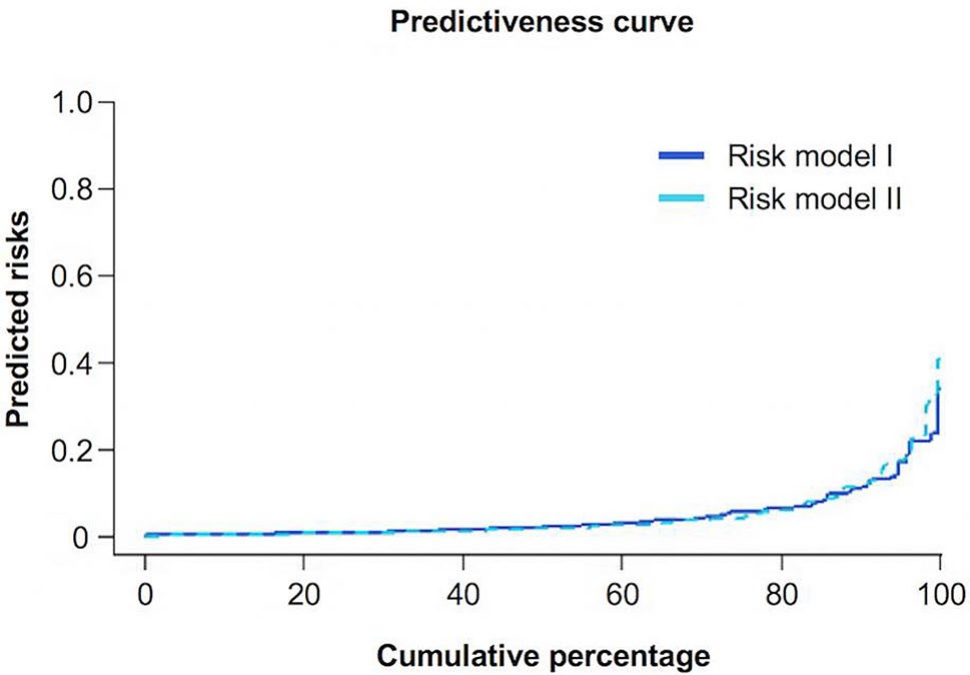
Predictiveness curves of both models. Predictiveness curves of Model I (without ethnicity) and Model II (with ethnicity) were almost identical

To evaluate the influence of ethnicity on in-hospital death, we first used the two different multiple logistic regression models (Table 2). The Hosmer–Lemeshow test for goodness of fit demonstrated no evidence of poor fit for Models I and II (*p* = 0.26 and = 0.39, respectively). The Brier score was 0.038 for Model I and 0.037 for Model II, demonstrating that these two models were nearly identical with respect to risk predictions. On ROC analysis, the area under the curve (AUC) (95% confidence interval) was 0.81 (0.78–0.86) for Model I and 0.83 (0.78–0.88) for Model II. The comparisons of ROC curves to test for statistically significant differences between the areas under the ROC curves AUCII and AUCI using Venkatraman and DeLong tests, demonstrate again that these two models had an identical classification performance ((AUCII—AUCI) = 0 for condition = in-hospital death; Venkatraman *p* = 0.15; Delong *p* = 0.052). The predictiveness curves of both models were also almost identical (Fig. 2). Both predictiveness curves calculated the risk of in-hospital death as ≤ 10% for around 90% of the study population.

**Table 2:** Two different models of multiple logistic regression Brier analysis and their scores

	Risk model I		Risk model II	
	Odds ratio	*p* value	Odds ratio	*p* value
	(95% CI)		(95% CI)	
Intercept	0.04 (0.01 to 0.12)	< 0.001	0.02 (0.01 to 0.09)	< 0.001
Female sex	0.54 (0.27 to 1.09)	0.09	0.68 (0.33 to 1.39)	0.29
Physical stress	3.79 (1.90 to 7.55)	< 0.001	3.48 (1.73 to 6.99)	0.001
Emotional stress	0.53 (0.16 to 1.69)	0.28	0.63 (0.19 to 2.03)	0.44
Age 51–74 yr	0.36 (0.14 to 0.91)	0.030	0.30 (0.12 to 0.77)	0.013
Age ≥ 75 yr	0.91 (0.38 to 2.21)	0.83	0.67 (0.27 to 1.68)	0.39
HR > 94 bpm	1.85 (1.06 to 3.22)	0.030	1.58 (0.89 to 2.78)	0.12
SBP > 140 mmHg	1.01 (0.55 to 1.84)	0.99	1.00 (0.55 to 1.85)	0.99
LVEF > 45%	0.56 (0.28 to 1.12)	0.10	0.51 (0.25 to 1.01)	0.06
WBC > 10 × 10^3^ cells/μL	2.25 (1.25 to 4.06)	0.007	2.35 (1.29 to 4.29)	0.005
Ethnicity (Japanese)	–	–	3.18 (1.76 to 5.73)	< 0.001
Brier score	0.038		0.037	

*Bpm* beats per minute, *CI*  confidence interval, *HR*  heart rate, *LVEF*  left ventricular ejection fraction, *SBP* systolic blood pressure, *WBC*  white blood cell count

**Fig. 3:**
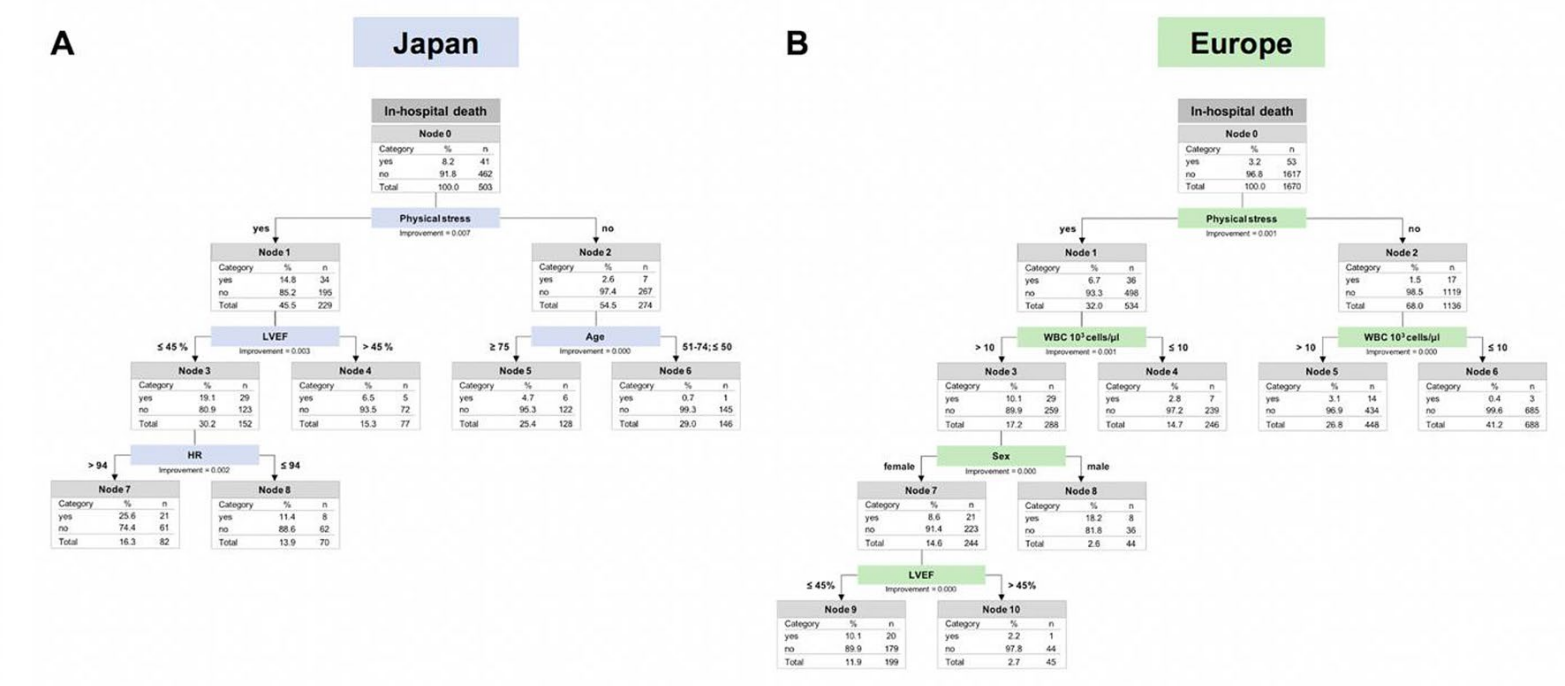
Decision trees. Decision trees for Japanese patients (**a**) and European patients (**b**). Presence of physical stressors was the most important prognostic factor for in-hospital mortality in the Japanese and European cohorts. The second most important prognostic factor was LVEF for Japanese patients, and the white blood cell count for European patients. *LVEF* left ventricular ejection fraction, *SBP* systolic blood pressure, *WBC* white blood cell count

Radial Basis Function Networks and CART were applied to analyze the effect of various variables on in-hospital death in the Japanese and European cohorts. The normalized importance of the respective variables for the Japanese and European cohorts is summarized in Supplementary Figs. 2 and 3. Physical stress was the most important prognostic factor for in-hospital mortality in both cohorts. The second most important prognostic factor was LVEF by both CART and RBF-nets for Japanese patients, while it was white blood cell count by CART or heart rate by RBF-nets for European patients. The in-hospital mortality rate in TTS patients without physical stress (*n* = 1410; 64.9%) was 1.7%, with no significant difference between the Japanese (*n* = 274) and European (*n* = 1136) cohorts (2.6 vs. 1.5%, *p* = 0.22) (Fig. 3).

## Discussion

This study aimed to investigate differences in clinical and demographic characteristics and in-hospital outcomes between Japanese and European TTS patients and to determine whether ethnicity affects outcomes. The main findings were as follows: (2) Japanese patients were more likely to have physical triggers, and less likely to have emotional triggers; (3) Japanese patients required more often catecholamines and/or IABP to maintain hemodynamic stability; and (4) Japanese patients had higher in-hospital mortality, although ethnicity per se did not appear to have an impact on the higher mortality.

No large-scale study has been conducted so far to compare TTS features between different ethnic groups. In a small study, Maekawa et al. found no significant difference in clinical characteristics between TTS patients in Japan (*n* = 12) and USA (*n* = 34) [17]. However, recent reports from Europe and Japan have demonstrated some differences in the demographic characteristics of those affected in these two regions: in Europe 85–90% of patients are women, and the mean age of patients is 65–70 years [13, 23, 24], whereas in Japan, 75–85% are women, and the mean age of patients is 70–75 years [25–27]. These findings were confirmed in the present study, the largest head-to-head comparison to date. However, it should be noted that these disparities may reflect potential differences in patient selection and the older age of the general population in Japan [28].

The present study found significant differences between the two cohorts in medications being used by the patients at admission. This difference may have partly affected in-hospital outcomes. Japanese patients were more likely to be on regular calcium-channel antagonists at the TTS index event. These drugs are obviously contraindicated in patients with acute heart failure because of their negative inotropic effect [29] and their activation of the sympathetic nervous system [30]. In contrast, European TTS patients were more likely to receive beta-blockers, however, which have failed to show a beneficial effect on 1-year mortality [13]. Regular treatment with beta-blockers before admission may have protected patients with apical ballooning from left ventricular outflow tract obstruction [25]. These disparities in drug prescription seem to be due to different guidelines for the treatment of hypertension in these two regions [29, 31].

From a clinical perspective, the most important finding of the present study was the unanticipated worse in-hospital outcome in Japanese patients. We demonstrated by machine learning approach that the prognostic factors for in-hospital mortality were almost same in both cohorts; however, the relative importance of the factors varied. Physical stress was the most important prognostic factor for in-hospital mortality in both cohorts. However, the prevalence of physical stressors was higher in Japanese patients and might have been responsible for the higher in-hospital mortality in this cohort. This observation is in line with our previous study demonstrating that physical triggers of TTS are independent predictors of poor in-hospital outcomes [12, 13]. Japanese patients were more likely to have cardiogenic shock than European patients. It is notable that in patients with cardiogenic shock IABP was more often used in Japan compared to Europe (35.9 vs. 23.5%).

Although a role for genetics in TTS has not yet been fully uncovered, we examined whether ethnicity independently influences outcomes. Brinjikji et al., in their nation-wide study in the US, found that ethnicity was not associated with in-hospital mortality in TTS [32]; their study, however, had a very small proportion of Asian patients and included only patients located in the US without taking also geographic differences into account. Notably, we revealed in the present study by use of three different statistical approaches that two different multiple logistic regression models (with and without ethnicity) had almost same accuracies of a risk prediction for in-hospital death. This observation can be interpreted that ethnicity, per se, does not have an additional impact on in-hospital death beyond that caused by differences of other clinical backgrounds. Nevertheless, patients with different ethnicities were enrolled from different regions, and so differences between groups could be due to geographic and socioeconomic factors and regional differences in therapeutic strategies. Therefore, further studies are needed to clarify the impact of genetic factors on in-hospital outcomes of TTS patients.

## Conclusions

This large-scale head-to-head comparative study provides important insights into ethnic disparities between Japanese and European TTS patients. Japanese patients are older and more likely to be male than European patients; they also have a significantly higher prevalence of physical triggers. Cardiogenic shock and in-hospital death are more common in Japanese patients. Ethnicity, per se, does not appear to have an additional impact on in-hospital mortality, beyond that caused by differences of other clinical backgrounds. Presence of physical stress is the most important prognostic factor in both Japanese and European patients. Thus, the worse in-hospital outcome in Japanese patients is mainly driven by the higher prevalence of physical triggers in this cohort.

## Supplementary Information

Below is the link to the electronic supplementary material.

Supplementary file1 (DOCX 64148 kb)
