# Supplementary material for: Ethnic comparison in takotsubo syndrome: novel insights from the International Takotsubo Registry
Source: Clin Res Cardiol. 2021 May 19;111(2):186–96. doi: 10.1007/s00392-021-01857-4 (PMC8816760; doi:10.1007/s00392-021-01857-4)
Supplement: Supplementary file 1 — Supplementary file1 (DOCX 64148 kb) [file 392_2021_1857_MOESM1_ESM.docx]

**Supplementary Appendix**

**Supplementary Figure 1. Triggering factors.**

**
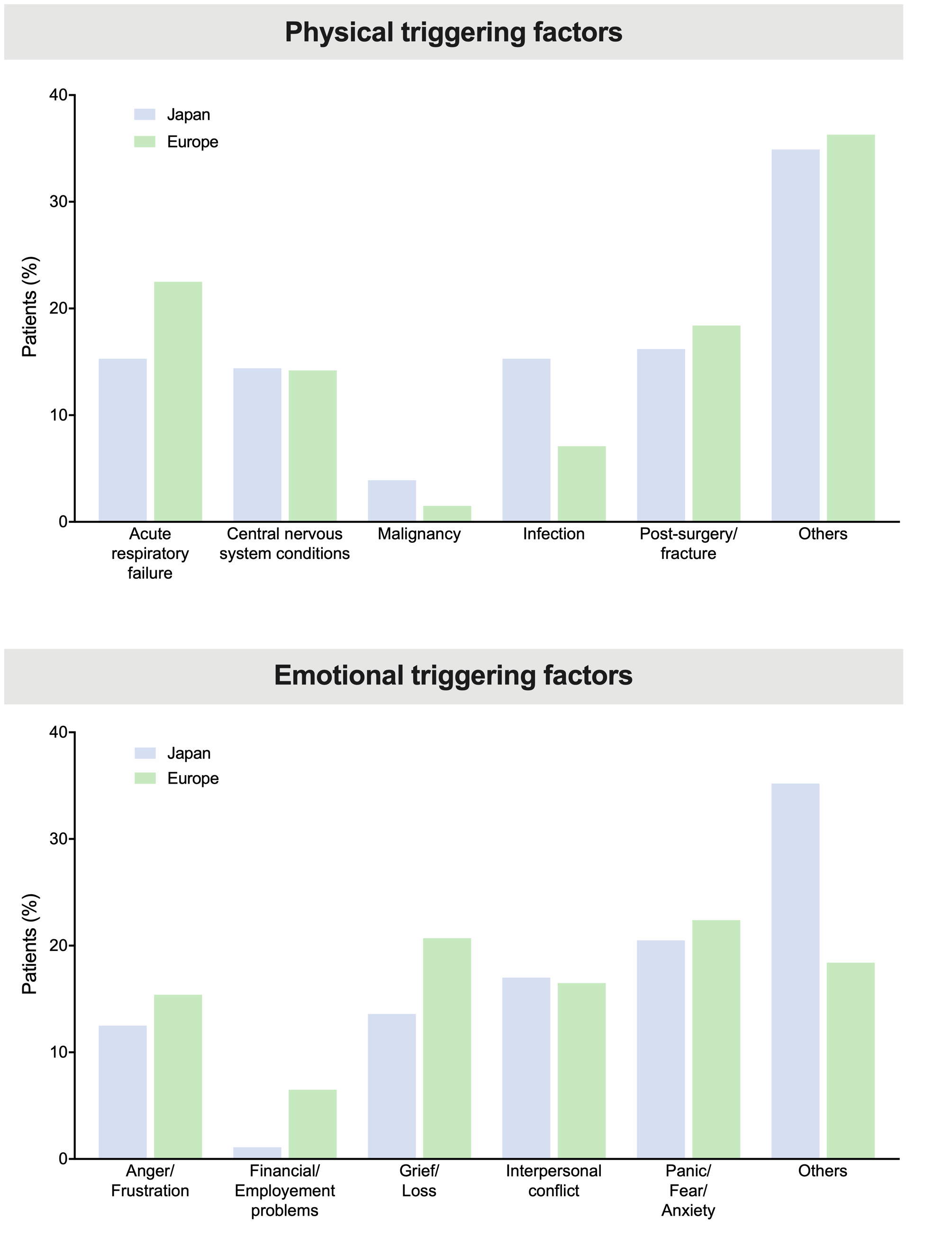
**

Physical and emotional triggering factors in Japanese and European patients.

**Supplementary Figure 2. Normalized importance of each input variable in a CART model.**

**
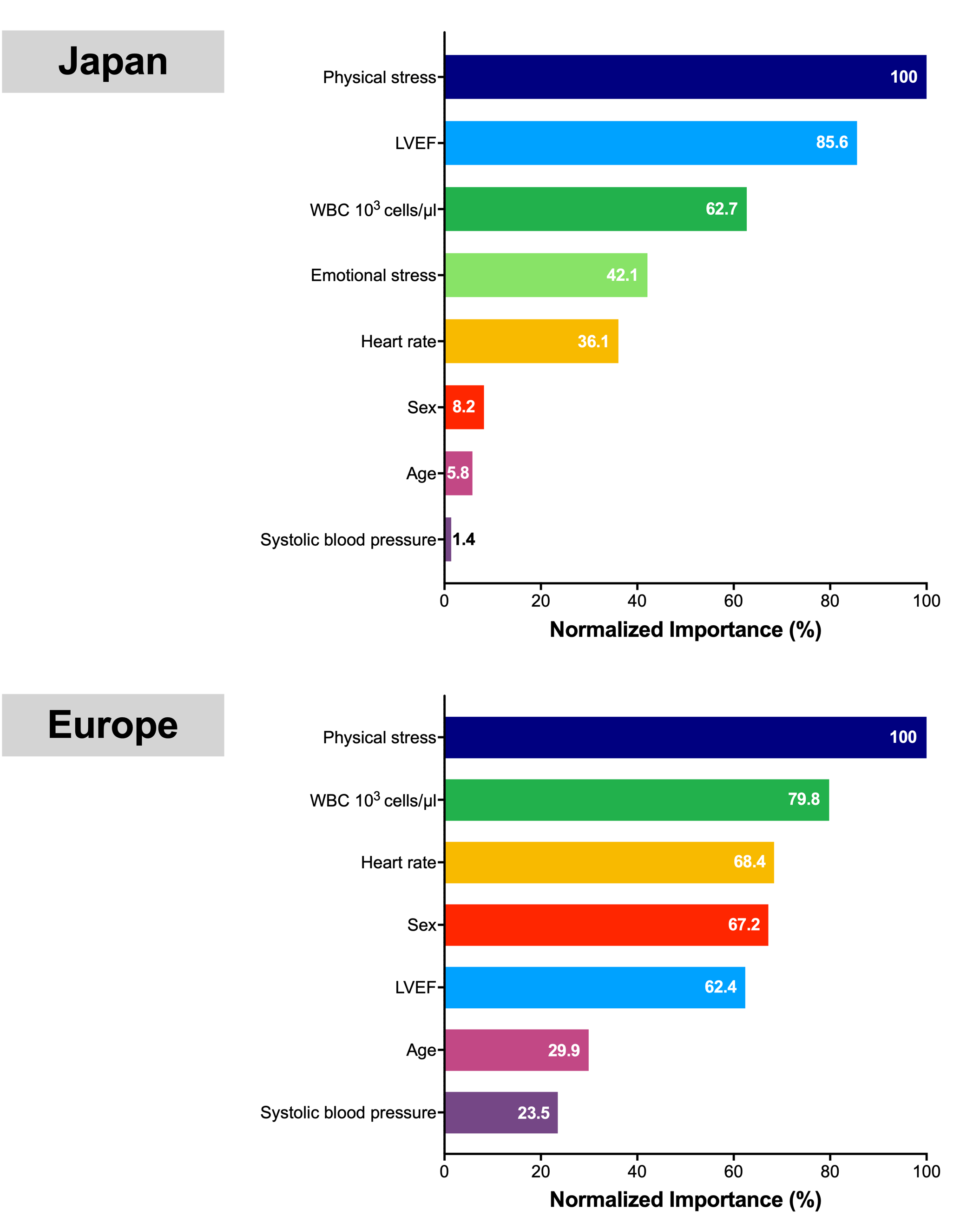
**

Classification and regression trees (CART) revealed that physical stress is the most important parameter for the prediction of in-hospital death both in Japan and Europe.

LVEF = left ventricular ejection fraction; WBC = white blood cell count.

**Supplementary Figure 3. Normalized importance of each input variable in a RBF-nets model.**


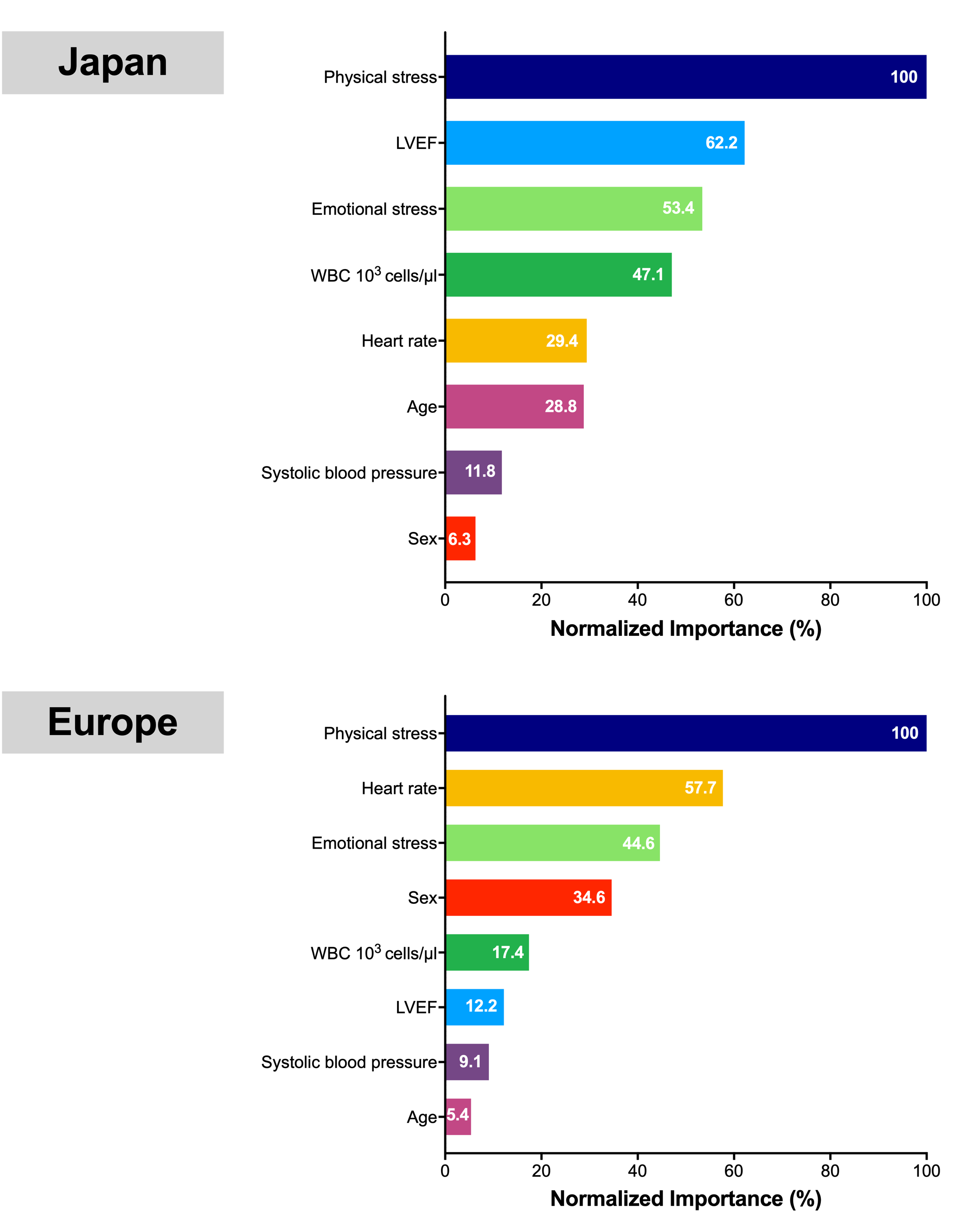


Radial basis function network (RBF-nets) revealed that physical stress is the most important parameter for the prediction of in-hospital death both in Japan and Europe.

LVEF = left ventricular ejection fraction; WBC = white blood cell count.
